# Supplementary material for: Regional impact of aging population on economic development in China: Evidence from panel threshold regression (PTR)
Source: PLoS One. 2023 Mar 14;18(3):e0282913. doi: 10.1371/journal.pone.0282913 (PMC10013891; doi:10.1371/journal.pone.0282913)
Supplement: S1 Appendix — (DOCX) [file pone.0282913.s002.docx]

# Appendix 1

Figure 1: The Old Dependency Ratio and Per Capita GDP of Beijing

Figure 2: The Old Dependency Ratio and Per Capita GDP of Tianjin

Figure 3: The Old Dependency Ratio and Per Capita GDP of Hebei

Figure 4: The Old Dependency Ratio and Per Capita GDP of Shanxi

Figure 5: The Old Dependency Ratio and Per Capita GDP of Mongolia

Figure 6: The Old Dependency Ratio and Per Capita GDP of Liaoning

Figure 7: The Old Dependency Ratio and Per Capita GDP of Jilin

Figure 8: The Old Dependency Ratio and Per Capita GDP of Heilongjiang

Figure 9: The Old Dependency Ratio and Per Capita GDP of Shanghai

Figure 10: The Old Dependency Ratio and Per Capita GDP of Jiangsu

Figure 11: The Old Dependency Ratio and Per Capita GDP of Zhejiang

Figure 12: The Old Dependency Ratio and Per Capita GDP of Anhui

Figure 13: The Old Dependency Ratio and Per Capita GDP of Fujian

Figure 14: The Old Dependency Ratio and Per Capita GDP of Jiangxi

Figure 15: The Old Dependency Ratio and Per Capita GDP of Shandong

Figure 16: The Old Dependency Ratio and Per Capita GDP of Henan

Figure 17: The Old Dependency Ratio and Per Capita GDP of Hubei

Figure 18: The Old Dependency Ratio and Per Capita GDP of Hunan

Figure 19: The Old Dependency Ratio and Per Capita GDP of Guangdong

Figure 20: The Old Dependency Ratio and Per Capita GDP of Guangxi

Figure 21: The Old Dependency Ratio and Per Capita GDP of Hainan

Figure 22: The Old Dependency Ratio and Per Capita GDP of Chongqing

Figure 23: The Old Dependency Ratio and Per Capita GDP of Sichuan

Figure 24: The Old Dependency Ratio and Per Capita GDP of Guizhou

Figure 25: The Old Dependency Ratio and Per Capita GDP of Yunnan

Figure 26: The Old Dependency Ratio and Per Capita GDP of Tibet

Figure 27: The Old Dependency Ratio and Per Capita GDP of Shanxxi

Figure 28: The Old Dependency Ratio and Per Capita GDP of Gansu

Figure 29: The Old Dependency Ratio and Per Capita GDP of Qinghai

Figure 30: The Old Dependency Ratio and Per Capita GDP of Ningxia

Figure 31: The Old Dependency Ratio and Per Capita GDP of Xinjiang
