# Supplementary material for: Regional impact of aging population on economic development in China: Evidence from panel threshold regression (PTR)
Source: PLoS One. 2023 Mar 14;18(3):e0282913. doi: 10.1371/journal.pone.0282913 (PMC10013891; doi:10.1371/journal.pone.0282913)
Supplement: Attachment — Submitted filename: Response to Reviewers.docx [file pone.0282913.s003.docx]

**Manuscript Title: Regional Impact of Aging Population on Economic Development in China: Evidence from Panel Threshold Regression (PTR)**

Dear Editors and Reviewers:

Thank you for providing us with the reviewers’ evaluation, comments and suggestions related to the paper entitled as above. We have made the corrections and modifications as commented and suggested. Furthermore, we believe those comments and suggestions will help us achieve more outstanding achievements in the future research.

Please find enclosed is the table prepared for the authors’ responses to the reviewers’ detailed comments. We sincerely hope this manuscript will finally be acceptable to be published in the ‘Plos One’ journal.

Thank you so much for all your help, and we look forward to hearing back from you soon.

Kind regards,

Authors of the manuscript entitled as above

**Authors’ Responses to Reviewers’ Comments**

| **No.** | **Comments from Reviewer(s)** | **Actions – All changes made in the manuscript are highlighted in red** | **Page** | **Paragraph** |
| --- | --- | --- | --- | --- |
| (1) | The paper has some potential, but it seems that the method rather than a scientific inquiry dominated the presented research. The presented methodology is rather sophisticated from the statistical point of view but has some serious flaws in the conceptual layer.    The last paragraph of the introduction should present, in a clear and explicit way, the aim of the article and briefly describe the structure of the article.  The literature review should be rewritten into regular sentences. Now it looks like a ppt bullet points pasted into an article. The authors write about numerous concepts and aspects but without clear reasoning; The authors should eliminate all ‘bullet-point sentences’ and rewrite those parts into regular sentences.  The review of previous research is very short, and has a form of a mechanistic and technical review only mentioning previous research rather than analysing their results and identifying the research gap. The review should be expanded and also foreign studies should be included | Thank you for the valuable comments.  In terms of the methodology, we have closely adapted and followed the following two published papers:  https://doi.org/10.1016/j.jclepro.2020.123773  <https://doi.org/10.1016/j.jclepro.2020.125381>  In addition, we have also referred to the following two published papers to ensure the accuracy and reliability of the methodology:  <https://doi.org/10.1016/j.spc.2021.06.029>  <https://doi.org/10.1016/j.ecolecon.2016.10.019>  For the coding of the Panel Threshold Regression in Stata, we have adapted and followed the study by Wang (2015):  <https://doi.org/10.1177/1536867X1501500108>  The aim and structure of the paper have been updated.  Literature review has been rewritten and hypotheses have been updated. | 5^th^  7^th^-10^th^ | 2^nd^ |
| (2) | It is a good effort. However, following minor changes are recommended before final publication:  1. Please remove the heading “5.1 Major Findings”. If you present conclusions here would be more appropriate.  2. Please check equation numbers after equation 11. Same number i.e., 1 is repeated erroneously  3. The paper contains many grammatical mistakes. It is recommended to get the paper language edited by any authentic native speaker.  4. Although, this journal encourages to publish replication studies but yet you need to highlight the research contributions. I have found the study contributions at the latter parts of literature review section. Please move them to introduction section.  5. Discussion part is not supported by past literature. Add more studies in this section that accept or negate your findings. Please read and cite the following papers carefully: <https://doi.org/10.3389/fenvs.2022.955744> <https://doi.org/10.3389/fenvs.2022.841380> <https://doi.org/10.1108/SAJBS-05-2020-0150> | Thank you for the valuable comments.  “Major Findings” has been removed and “Conclusions” has been presented as suggested.  Equation numbers have been corrected.  Grammatical mistakes have been modified by the proofreader.  We have deleted the contributions at the latter parts of literature review section and the contributions have been rewritten at the latter parts of introduction section.  Past literatures and following papers have been added. | 23^rd^  9^th^ and 10^th^  5^th^  20^th^, 22^nd^ and 26^th^ | 1^st^ |
| Additional Editor Comments | I have read your manuscript “Regional Impact of Aging Population on Economic Development in China” and I found many positive things in it. However, in my opinion the paper has some shortcomings in regards to text, Below I have provided numerous remarks on the text:  1. In several instances, I suggest to cite more relevant and recent studies in the introduction and methodology sections.  2. The introduction should be expanded to include importance, uniqueness and contribution to the literature. The description of the contribution needs to be more forensic, needs to be more focused.  3. You need to add limitations of the study. | Thank you for the valuable comments.  More relevant and recent studies have been added in the introduction and methodology sections.  The importance, uniqueness, and contribution have been added in the introduction.  Limitations have been added at the latter parts of Conclusions remarks and implications. | 3^rd,^ 4^th^, and 5^th^  5^th^  25^th^ |  |
